# Biofilm Formation in *Arcobacter butzleri* and *Arcobacter cryaerophilus*: Phenotypic and Genotypic Characterization of Food and Environmental Isolates

**DOI:** 10.3390/microorganisms13122835

**Published:** 2025-12-13

**Authors:** Irena Musilová, Kateřina Kozlová, David Šilha

**Affiliations:** Department of Biological and Biochemical Sciences, Faculty of Chemical Technology, University of Pardubice, Studentská 573, 532 10 Pardubice, Czech Republic

**Keywords:** *Arcobacter butzleri*, *Arcobacter cryaerophilus*, biofilm-associated genes, biofilm formation, dynamic biofilm assay, foodborne pathogen, static biofilm assay

## Abstract

*Arcobacter butzleri* and *Arcobacter cryaerophilus* are emerging foodborne and waterborne pathogens associated with enteritis and extraintestinal infections in humans. Their persistence in the environment and resistance to antimicrobial treatment are closely related to their ability to form biofilms, which provide protection against adverse conditions and support survival on food contact surfaces. This study evaluated both the genotypic and phenotypic aspects of biofilm formation among *A*. *butzleri* and *A*. *cryaerophilus* isolates from food and environmental sources. Six biofilm-associated genes (*flaA*, *flaB*, *fliS*, *luxS*, *pta*, and *spoT*) were detected by multiplex PCR, and biofilm production was assessed using the Christensen microtiter plate assay and Congo Red Agar (CRA) test. All *A*. *cryaerophilus* isolates carried the same gene set as *A*. *butzleri*, suggesting conserved genetic determinants of motility and *Quorum sensing*. However, phenotypic assays revealed interspecific variability: while most *A*. *butzleri* isolates formed strong biofilms, 70% of *A*. *cryaerophilus* strains showed moderate to strong formation despite all being CRA-negative. No direct correlation between gene presence and biofilm intensity was observed, indicating complex regulation of biofilm development. This study provides a comparative overview of biofilm formation in *A*. *butzleri* and *A*. *cryaerophilus* and highlights their adaptive potential and persistence in food-related environments.

## 1. Introduction

*Arcobacter butzleri* has emerged as an important zoonotic and foodborne Gram-negative pathogen, frequently isolated from raw meat, poultry, milk, and environmental water sources. It has been associated with a range of human diseases, including gastroenteritis, bacteremia, and septicemia, particularly in immunocompromised individuals [1,2]. The increasing detection of *Arcobacter* in food production environments highlights its remarkable environmental persistence and resistance to disinfection procedures [3,4]. Moreover, several studies have reported resistance of *A*. *butzleri* and *A*. *cryaerophilus* strains to multiple classes of antimicrobials, including macrolides, fluoroquinolones, and β-lactams, which complicates clinical management and may reflect adaptive mechanisms that enhance virulence [4,5,6].

A key factor contributing to the environmental persistence and antimicrobial resistance of *Arcobacter* is its ability to form biofilms. Biofilms provide a structural and physiological advantage, enabling bacterial cells to survive under nutrient-limited or stress conditions, resist sanitizing agents, and evade host immune responses [7,8]. Thus, biofilm formation is considered a crucial virulence trait supporting both environmental survival and host infection. Biofilm formation in *A*. *butzleri* and *A*. *cryaerophilus* has been documented on various abiotic surfaces such as stainless steel, glass, and polystyrene, highlighting its potential role in cross-contamination during food processing [4,9,10]. Biofilm formation is a multistage process involving initial adhesion, maturation, and dispersion, regulated by motility, *Quorum sensing*, stress response, and metabolic pathways. Environmental factors such as atmospheric conditions, nutrient availability and medium composition strongly influence motility and subsequent biofilm development [11]. Additionally, host-associated factors (e.g., bile salts) have been demonstrated to modulate both motility and biofilm formation in *A. butzleri*, highlighting the role of gastrointestinal conditions in virulence expression [12].

Several genes have been proposed to contribute to biofilm development in *A*. *butzleri* or *A*. *cryaerophilus* and related *Campylobacter* species, including *flaA*, *flaB*, *fliS*, *luxS*, *pta*, and *spoT* [13]. The *flaA* and *flaB* genes encode structural flagellin subunits that are essential for motility and initial surface attachment. Mutations in these genes reduce motility and adhesion, impairing biofilm formation and demonstrating that flagellar function is critical for both locomotion and surface colonization [13,14]. The *fliS* gene encodes a flagellar chaperone involved in the correct folding and export of flagellin proteins; its disruption similarly reduces motility and biofilm biomass [15]. The *luxS* gene participates in the production of the autoinducer-2 (AI-2) signaling molecule, a key component of the *quorum-sensing* system that coordinates population-level behaviors, including biofilm maturation and virulence factor expression [8,16]. Mutants lacking luxS exhibit significantly reduced biofilm formation, supporting the importance of *Quorum sensing* in regulating multicellular behavior in *A*. *butzleri* [13].

The *pta* gene encodes phosphate acetyltransferase, a key enzyme in the Pta-AckA pathway linking central metabolism and acetyl-phosphate signaling. Its role in biofilm formation is likely related to metabolic regulation and surface-dependent responses, as pta mutants exhibit variable biofilm production depending on environmental conditions [15]. Finally, the *spoT* gene encodes a bifunctional enzyme involved in the synthesis and degradation of (p)ppGpp, a global regulator of the stringent response. This molecule mediates adaptation to nutrient limitation and stress, influencing the transcription of genes related to motility, adhesion, and virulence [13,17]. In *A*. *butzleri*, mutations in *spoT* lead to surface-specific changes in biofilm formation, suggesting that the stringent response fine-tunes biofilm development in response to environmental cues.

Together, these genes constitute interconnected regulatory and structural systems that enable *A*. *butzleri* to form biofilms, persist under adverse conditions, and potentially resist antimicrobial therapy. Understanding the molecular basis of biofilm formation, particularly the roles of *flaA*, *flaB*, *fliS*, *luxS*, *pta*, and *spoT*, is therefore critical for elucidating mechanisms underlying virulence, persistence, and resistance in both food-processing and clinical contexts [13,15].

However, a comprehensive assessment of biofilm formation requires integrating both genotypic and phenotypic approaches. While the detection of biofilm-associated genes provides valuable insights into the potential of a strain to form biofilms, it does not necessarily reflect the actual biofilm-forming ability under specific environmental or physiological conditions [8]. Phenotypic evaluation through quantitative and qualitative assays remains essential to verify gene expression and understand the influence of environmental factors on biofilm development. Therefore, simultaneous investigation of biofilm-associated genes and observable biofilm formation enables a more accurate characterization of *A*. *butzleri* and *A*. *cryaerophilus* strains and contributes to a deeper understanding of their ecological fitness and pathogenic potential.

The aim of this study was to evaluate the role of selected biofilm-associated genes in *Arcobacter* and to determine their influence on strain-specific biofilm-forming ability. Notably, this work provides a novel perspective by examining biofilm formation under both static and dynamic conditions—an aspect that has received limited attention in previous studies. This dual approach allows a more comprehensive assessment of *A. butzleri* and *A*. *cryaerophilus* capacity to adapt to diverse environmental settings. Furthermore, additional phenotypic traits potentially linked to biofilm formation, including motility and colony morphology on Congo Red agar, were also investigated.

## 2. Materials and Methods

### 2.1. A. butzleri Strains Identification by mPCR and 16S rRNA-RFLP

A total of 60 *Arcobacter* strains were recovered from food and environmental water samples following previously established protocols [18]. Prior to experimental use, isolates were cultured on tryptone soya agar (TSA; HiMedia, Mumbai, India) for 48 h at 30 °C under aerobic conditions. Cells were subsequently harvested and suspended in physiological saline to a turbidity equivalent to 0.5 McFarland (~1.5 × 10^8^ CFU/mL). Suspensions were further diluted as needed for downstream analyses, and actual cell concentrations were verified by plating a presumptive 10^3^ CFU/mL dilution on TSA.

Strain identification was performed using multiplex PCR (*m*PCR) as described previously [10,19], with minor modifications. Each PCR reaction was carried out in a total volume of 50 µL containing PCR-grade water, 2.5 µL of 10× PCR buffer, 1.5 U *Taq* DNA polymerase, and *d*NTP mixture at a final concentration of 0.2 mM each. The reaction mix was supplemented with 1.5 mM MgCl_2_ and 50 pmol of each primer: *ButR*, *SkiR*, *TherR*, *CibR*, *ArcoF*, *GyrasF*, and *GyrasR*. Thermal cycling consisted of an initial denaturation followed by 30 cycles of 94 °C for 45 s (denaturation), 58 °C for 45 s (annealing), and 72 °C for 2 min (extension). Amplification products were separated on a 2% agarose gel in 0.5× TBE buffer at 120 V for 2 h.

Due to the possibility of distinguishing *A*. *cryaerophilus* strains (1A and 1B) and also to increase the reliability of identification based on previous knowledge, all isolates were additionally analyzed using 16S *r*RNA restriction fragment length polymorphism (RFLP) [10,20]. Genomic DNA served as a template for amplification of a 1026 bp fragment of the 16S *r*RNA gene [21]. Amplicons were digested with *Mse*I or *Mn*II*/Bfa*I (New England Biolabs, Ipswich, MA, USA) in a 25 µL reaction containing 0.5 µL of PCR product, 5 U of the respective restriction enzyme, 2.5 µL of 10× CutSmart buffer, and PCR-grade water. Digestions were performed at 37 °C for 15 min (*Mse*I, *Mn*II) or 1 h (*Bfa*I), followed by enzyme inactivation at 65 °C for 20 min (*Mse*I, *Mn*II) or at 80 °C for 20 min (*Bfa*I) according to the manufacturer’s instructions. Resulting fragments were resolved on 3% agarose gels in 1× TBE buffer at 100 V for 2 h. A 50 bp DNA ladder (BiotechRabbit, Berlin, Germany) was used as a molecular size marker. Gels were stained with ethidium bromide and visualized using a UV transilluminator.

### 2.2. Biofilm-Associated Gene Detection

The presence of six biofilm-associated genes (*flaA*, *flaB*, *fliS*, *luxS*, *pta*, and *spoT*) was determined according to a previous study [13] using two multiplex PCR (*m*PCR) assays. Each PCR reaction was performed in a final volume of 25 µL containing 2.5 µL of 10× PCR buffer (Mg^2+^-free), 3 mM Mg^2+^, 0.1 mM of each *d*NTP, 0.1 µM of each primer, 1.25 U of *Taq* DNA polymerase, and 2 µL of extracted template DNA. All PCR reagents were purchased from TaKaRa Bio Inc. (Japan). The following primer pairs were used for *A*. *butzleri* strains [15] were used: *fliS*-F (5′-AAAAGTGCAATACAAGAGGGTGA-3′) and *fliS*-R (5′-AGCAACATCTCCACCATCAAAA-3′) producing a 114 bp fragment; *flaA*-F (5′-CCAGCTGACATTTTAGCACCAC-3′) and *flaA*-R (5′-CTGCTGCAAGAACTGCAAAAGG-3′) yielding a 145 bp product; *luxS*-F (5′-TATTAGATAGTTTTAGAGTTGA-3′) and *luxS*-R (5′-TAAAATCCAGTTCTACAACCCAT-3′) yielding a 256 bp fragment; *flaB*-F (5′-TGCGTATGACCCTGCTTGTG-3′) and *flaB*-R (5′-CTGCTGCAAGAACTGCATTAGG-3′) producing a 419 bp product; *pta*-F (5′-AGATTTTTTGGTTGTATGATGGTAAGACT-3′) and *pta*-R (5′-GCAGCATCAGCTTGAAGCTCACCATCAAA-3′) yielding a 431 bp amplicon; and *spoT*-F (5′-TTGCCAATGAGCCGCAATTC-3′) and *spoT*-R (5′-AGCGGTGAACCTTACTGTGT-3′) producing a 936 bp product.

For *A. cryaerophilus*, the following primer pairs were used: *fliS*-F (5′-AAAAGTGCAATACAAGAGGGTGA-3′) and *fliS*-R (5′-AGCAACATCTCCACCATCAAAA-3′) producing a 335 bp fragment; *flaA*-F (5′-CCAGCTGACATTTTAGCACCAC-3′) and *flaA*-R (5′-CTGCTGCAAGAACTGCAAAAGG-3′) yielding a 78 bp product; *flaB*-F (5′-TGCGTATGACCCTGCTTGTG-3′) and *flaB*-R (5′-CTGCTGCAAGAACTGCATTAGG-3′) yielding a 182 bp product; and *spoT*-F (5′-TTGCCAATGAGCCGCAATTC-3′) and *spoT*-R (5′-AGCGGTGAACCTTACTGTGT-3′) producing a 275 bp product. Primer pairs for the genes *luxS* and *pta* in *A. cryaerophilus* were identical to those used for *A. butzleri*.

The PCR cycling conditions consisted of an initial denaturation at 94 °C for 3 min, followed by 30 cycles of denaturation at 94 °C for 30 s, primer annealing at 52 °C for 30 s (optimized within the range of 48–59 °C), and extension at 72 °C for 60 s. A final extension step was carried out at 72 °C for 10 min. *Arcobacter butzleri* LMG 10828 and *A*. *cryaerophilus* CCM 3933 was used as a positive control for all six target genes [13,15]. Amplified PCR products were separated on a 2% agarose gel containing ethidium bromide, using a 155–970 bp DNA ladder (Top Bio, Czech Republic) as a molecular size marker. Visualization of the PCR products was performed under UV illumination using a transilluminator (Chemos, Cítov, Czech Republic).

### 2.3. Motility Assay

Motility of each strain was evaluated by stab-inoculating single colonies into thioglycolate semisolid agar (0.4% agar; Himedia, Mumbai, India) as described previously [13]. Plates were incubated aerobically at 30 °C for 48 h. Motility was assessed by measuring the diameter of the motility zone, and experiments were performed in at least three independent replicates. All experiments were carried out in at least three independent replicates.

### 2.4. Congo-Red Assay

For each strain, 10 µL of a bacterial cell suspension (10^8^ CFU/mL) was applied onto the surface of Congo Red Agar (CRA) plates. CRA was composed of 37 g/L Brain Heart Infusion broth, 10 g/L agar, and 0.1 g/L sterile Congo red solution (CAS: 573-58-0; Acros Organics, Geel, Belgium) [22,23]. Plates were incubated at 30 °C for 48 h, after which colony color was assessed. Strains with red colonies were considered cellulose producers, while white or pale colonies were classified as non-producers. All experiments were performed in at least three independent replicates.

### 2.5. Biofilm Formation Assay

Biofilm formation was monitored in flat-bottomed 96-well microtiter plates (SPL Life Sciences, Pocheon-si, Republic of Korea) as previously described [10,24,25] with minor modifications. Briefly, 100 µL of bacterial cell suspension (10^7^ CFU/mL) in brain heart infusion broth (BHI; Himedia, Mumbai, India) was inoculated into each well. After incubation under specific conditions, wells were thoroughly rinsed five times with sterile distilled water and allowed to dry. Biofilms were fixed with 2% sodium acetate for 15 min, stained with 1% crystal violet (Sigma-Aldrich, St. Louis, MO, USA) for 15 min, washed, dried, and solubilized in 96% ethanol. Absorbance was measured at 595 nm using a microplate reader (Infinite M200, Tecan, Zurich, Switzerland). Biofilm formation of the *Arcobacter* strains was categorized according to a previously described system [25]. Each experiment included 24 wells, and all experiments were repeated independently three times.

### 2.6. Statistical Analysis

The obtained values were statistically evaluated using Excel 2016 (Microsoft, Redmond, WA, USA) and Statistica 12 (StatSoft, Tulsa, OK, USA). Extreme values were tested with the Dean-Dixon test and excluded with 95% probability. Median and standard deviation were determined from the remaining values. Potential errors from insufficient dye washing or unusually high absorbance were also considered and excluded. Significant differences were established at *p* values of <0.05.

## 3. Results

### 3.1. Detection of Biofilm-Associated Genes

The presence of genes potentially associated with biofilm formation was investigated in all 50 *A. butzleri* strains and 10 *A*. *cryaerophilus* strains, and the results are summarized in Table 1 and Table 2. Due to the number of targeted genes and the similar size of some PCR amplicons, amplification was divided into two separate reactions: one targeting *flaA*, *flaB*, and *spoT* and the other targeting *fliS*, *luxS*, and *pta*.

All 50 (100%) *A. butzleri* isolates were positive for the *fliS*, *luxS*, *pta*, and *spoT* genes. In contrast, *flaA* and *flaB* genes were either both present or both absent, which occurred in 15 (30%) strains. Interestingly, even strains lacking both *flaA* and *flaB* exhibited motility when tested in semisolid thioglycolate medium, suggesting that alternative mechanisms or compensatory motility-associated genes may contribute to flagellar function.

All 10 *A. cryaerophilus* isolates carried *fliS*, *luxS*, *pta*, *spoT*, *flaA*, and *flaB* genes. Unlike *A. butzleri*, no strain lacked *flaA* or *flaB*, indicating a conserved presence of flagellar and biofilm-associated genes across these isolates.

### 3.2. Bacterial Motility Assessed by Motility Assay

**Figure 1:**
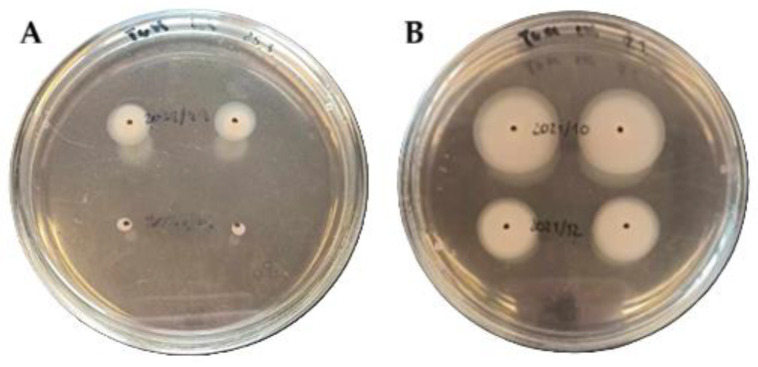
Representative motility phenotypes of *Arcobacter butzleri* strains. Panel (**A**) shows strains with low motility: UPCE 2021/49 (upper) and UPCE 2021/41 (lower). Panel (**B**) shows strains with moderate motility: UPCE 2021/10 (upper) and UPCE 2021/12 (lower).

Motility, expressed as the mean diameter of the motility zone on nutrient medium, is presented in Table 1 and Table 2. All 50 *A*. *butzleri* strains were actively motile, with zones ranging from 0.2 to 2.15 cm. According to previous study, motility was classified as low (<2.0 cm), moderate (2.0–4.0 cm), or high (>4.0 cm) [11]. Based on this classification, 48 strains (96%) displayed low motility, while 2 strains (4%) exhibited moderate motility. Representative phenotypic appearances of these two motility groups are presented in Figure 1.

In contrast, all *A. cryaerophilus* strains exhibited low motility, with zone diameters ranging from 0.4 to 1.5 cm. Despite the universal presence of *flaA* and *flaB*, their motility remained uniformly low, suggesting species-specific differences in flagellar regulation or expression. Overall, motility was generally more restricted in *A. cryaerophilus* compared to *A. butzleri*.

### 3.3. Biofilm Formation Assessed by Congo Red Agar (CRA) Assay

**Figure 2:**
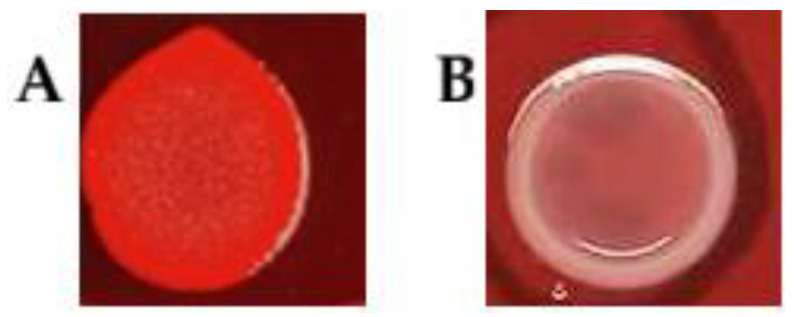
Pigmentation of *Arcobacter butzleri* colonies on CRA medium. Panel (**A**) shows strain UPCE 2021/11 exhibiting a red colony phenotype, while Panel (**B**) shows strain UPCE 2015/15 exhibiting a white colony phenotype.

Biofilm-forming potential was initially evaluated using Congo Red Agar as a phenotypic indicator of cellulose production. Only 5 strains (10%) of *A*. *butzleri* were identified as cellulose producers (biofilm-positive; red phenotype), whereas 45 strains (90%) were classified as biofilm-negative (white phenotype). Results are summarized in Table 1, while Figure 2 presents representative images illustrating these pigmentation phenotypes on CRA medium.

All *A. cryaerophilus* strains exhibited a white CRA phenotype (Tabel 2), consistent with the majority of *A. butzleri* isolates. This indicates that cellulose-based biofilm formation may be limited in these *Arcobacter* strains, with only a small fraction of *A. butzleri* exhibiting the red phenotype.

### 3.4. Quantitative Biofilm Formation Assessed by Christensen Method

Biofilm formation of the examined *A*. *butzleri* and *A*. *cryaerophilus* strains was quantitatively assessed using the Christensen colorimetric microtiter plate assay under both static and dynamic conditions. In *A*. *butzleri*, the majority of strains (96%) demonstrated the ability to form biofilms on a polystyrene surface. Only two *A*. *butzleri* strains (4%) were biofilm-negative, both under static and dynamic cultivation conditions. Under static conditions, 11 strains (22%) were classified as weak biofilm producers, 18 strains (36%) as moderate producers, 19 strains (38%) as strong producers, and only 2 strains (4%) were biofilm-negative. Under dynamic cultivation conditions, 13 strains (26%) were weak biofilm producers, 16 strains (32%) moderate producers, 17 strains (34%) strong producers, and 4 strains (8%) were classified as biofilm-negative. Seventeen strains (34%) consistently exhibited strong biofilm formation under both static and dynamic conditions. Among strains classified as weak producers under static conditions, most either retained this classification or showed biofilm-negativity under dynamic conditions. Only two strains demonstrated increased biofilm formation under dynamic cultivation. Overall, a higher level of biofilm formation was observed in 40 *A*. *butzleri* strains (80%) under static cultivation, although only 10 of these strains (25%) showed an increase sufficient to reclassify them into a higher biofilm intensity category. Conversely, 10 strains (20%) exhibited higher biofilm formation under dynamic cultivation (Table 1).

In *A. cryaerophilus*, biofilm formation was generally lower compared to *A. butzleri*. Under static conditions, 3 strains (30%) were weak producers, 4 (40%) moderate, and 3 (30%) strong, with no biofilm-negative isolates. Under dynamic conditions, 4 strains (40%) were weak, 3 (30%) moderate, and 3 (30%) strong producers. Overall, *A. cryaerophilus* showed less pronounced enhancement under dynamic cultivation compared to *A. butzleri*, indicating that dynamic conditions had a smaller impact on biofilm intensity in this species.

## 4. Discussion

The ability to form biofilms is widely acknowledged as a key determinant of microbial pathogenicity and persistence in both clinical and environmental contexts [26,27,28]. In many pathogenic and opportunistic bacterial species, biofilm formation has been associated with increased antimicrobial resistance, enhanced survival under stress, and elevated virulence [7,29]. Although biofilm formation has been reported in several *Arcobacter* species, including *A. butzleri* and *A. cryaerophilus*, the extent and mechanisms underlying this phenotype may differ between them [10,13,30]. Investigations into the specific genetic determinants of this phenotype in *A. butzleri* remain scarce, and even fewer studies have addressed *A. cryaerophilus* in this context. Moreover, few studies have examined biofilm formation under dynamic cultivation conditions that better mimic real-world environments.

In this study, we undertook a combined genotypic and phenotypic assessment of 50 food and environmental isolates of *A. butzleri* and 10 isolates of *A*. *cryaerophilus*, with the aim of elucidating the prevalence of six genes (*flaA*, *flaB*, *fliS*, *luxS*, *pta*, and *spoT*) previously linked with biofilm formation in *Campylobacter* spp. and other bacteria [13,31]. The phenotypes evaluated included (i) biofilm formation quantified by the Christensen colourimetric assay in microtiter plates [25], (ii) colony morphology on Congo-red agar (CRA) as an indicator of extracellular polysaccharide/cellulose production [22,23], and (iii) motility in semisolid thioglycolate medium.

Motility is a well-recognised virulence factor and a major contributor to initial surface adherence and biofilm initiation [32,33]. While *Arcobacter* spp. are generally motile by virtue of a polar flagellum [4,11,13,15], their motile activity is considered moderate compared with highly motile pathogens [13]. In our dataset, all 50 *A. butzleri* isolates were motile, yet 96% were classified as having low motility and 4% as moderate. Similarly, all *A. cryaerophilus* isolates were motile but exhibited uniformly low motility, suggesting that although both species are flagellated, their motility efficiency may differ due to species-specific regulation or flagellar structure [34]. Notably, atmospheric conditions and medium composition strongly influenced motility, as decreased motility of *A. butzleri* under microaerophilic compared with aerobic conditions has been documented [11].

Genotypically, all *A*. *butzleri* isolates (100%) harboured *fliS*, *luxS*, *pta*, and *spoT*, whereas the two flagellar genes *flaA* and *flaB* were detected together in only 15 (30%) strains. The tight co-occurrence of *flaA* and *flaB* suggests functional linkage, given that both encode components of the flagellar filament essential for motility and adherence [35]. In contrast, all *A. cryaerophilus* isolates carried the full set of targeted genes, including *flaA* and *flaB*, which may reflect lower genomic variability within this species. Interestingly, however, the presence of these genes did not strictly correlate with motility phenotypes: some *flaA*/*flaB*-negative isolates were motile, while some gene-positive isolates exhibited weak or absent motility. This decoupling between gene presence and phenotype, observed in both species, underscores the role of post-transcriptional and regulatory mechanisms influencing motility in *Arcobacter* spp. This discrepancy aligns with findings in *Arcobacter* and related genera, where *flaA* disruption abolished motility whereas *flaB* mutants retained motility [34]. Environmental regulation of flagellar expression and low sequence homology of filament subunits among strains may explain this variability [13,34].

The CRA method, based on Congo red binding to curli, fimbriae and cellulose, is used as a rapid phenotypic screen of biofilm-matrix production [22,23]. While employed in many bacteria (e.g., *Escherichia coli*, *Klebsiella pneumoniae*, *Campylobacter jejuni*, *Staphylococcus* spp.) [13,36,37], its efficacy for *Arcobacter* spp. has been under-studied. In our work, CRA phenotypes did not correlate with quantitative biofilm production by Christensen assay, e.g., isolates *A. butzleri* UPCE 2021/47, *A*. *butzleri* UPCE 42 and *A*. *butzleri* UPCE 24B produced high biofilm (OD 0.2830–0.4073) but exhibited white (negative) phenotype on CRA. Conversely, some low biofilm-producers (e.g., *A*. *butzleri* UPCE 93) showed red CRA phenotypes. Only 10% of isolates were classified as biofilm-positive by CRA, while 96% showed biofilm formation by microtiter assay. All *A. cryaerophilus* isolates displayed white CRA phenotypes, confirming limited cellulose production in this species and suggesting that the CRA test is generally unsuitable for differentiating biofilm potential within *Arcobacter*. Our findings are consistent with previous studies, which concluded that CRA is not a reliable indicator of biofilm production in *A. butzleri* [13].

Biofilm formation on abiotic surfaces remains a critical challenge across sectors including food production, healthcare and water systems [38,39]. For *A. butzleri*, prior studies report wide ranges of biofilm-positivity (21–32% up to >90%) depending on methodology and isolate origin [8,13,38]. Our data show that *A. cryaerophilus* isolates also exhibit considerable biofilm potential, though typically lower in intensity than *A. butzleri*, with the majority classified as weak to moderate producers under both static and dynamic conditions. The slightly reduced biofilm formation under agitation parallels observations in other bacteria; e.g., Stepanović et al. [40] showed decreased biofilm formation of *Staphylococcus epidermidis* and *Salmonella* spp. under dynamic cultivation. These results together indicate that hydrodynamic shear interferes with initial adhesion and microcolony formation in both *A. butzleri* and *A. cryaerophilus*.

The intensity of biofilm formation also significantly depends on strain origin and environmental conditions [13,30]. Girbau et al. [38] reported higher biofilm formation under aerobic conditions for *A. butzleri*, while Martins et al. [11] observed increased biofilm formation under microaerophilic atmosphere. Comparable species-specific responses have been observed for *A. cryaerophilus*, which appears more sensitive to oxygen limitation, exhibiting better growth but weaker biofilm development under microaerophilic conditions [3,10]. Surface material affects adherence: *A. butzleri* shows high affinity to polystyrene, borosilicate glass and stainless steel common in food-processing facilities [10,15,38]. Temperature, incubation time and cell density further modulate biofilm development [13].

While the genetic basis of biofilm formation is well characterised in other bacteria [41], knowledge for *Arcobacter* remains limited. We focused on six genes—*flaA*, *flaB*, *fliS*, *luxS*, *pta*, and *spoT*—previously implicated in motility, *Quorum sensing*, energy metabolism and stress response [13,15]. In all *A*. *butzleri* isolates (100%) *fliS*, *luxS*, *pta* and *spoT* were present, whereas *flaA* and *flaB* were present together in only 30%. By contrast, all *A. cryaerophilus* isolates carried these six loci, yet their biofilm phenotype varied from weak to strong, supporting the conclusion that gene presence alone is not predictive of phenotype within *Arcobacter*. Despite universal presence of these loci, phenotypic biofilm capacity remained highly variable, suggesting that gene presence alone is insufficient to predict phenotype, and that gene expression, regulation and strain-specific background are important [13,15].

Notably, absence of *flaA*/*flaB* was generally associated with weaker biofilm formation, in line with the hypothesis that flagella support initial adhesion [13]. Presence of *spoT* was positively associated with moderate or strong biofilm formation—*spoT* encodes a (p)ppGpp synthetase/hydrolase, a central enzyme in the stringent response that may enhance cell aggregation under stress [42]. The *pta* gene, encoding phosphotransacetylase, also showed positive association with biofilm formation (77% of *pta*-positive isolates produced moderate/strong biofilms), consistent with findings that energy-intensive biofilm formation depends on acetate metabolism [4,8,43,44]. In contrast, the role of *luxS*, central to AI-2 *quorum*-*sensing*, was ambiguous: its absence corresponded to slightly reduced biofilm formation in our work, but without statistical significance—similar ambiguity has been reported in *K. pneumoniae* and *S. gordonii* [45,46]. The consistent presence of *luxS* in *A. cryaerophilus* but variable biofilm intensity suggests that interspecies differences in *quorum*-*sensing* regulation may underlie the observed variation between *Arcobacter* species.

Our results highlight that biofilm formation in *A. butzleri* and *A*. *cryaerophilus* is a multifactorial, strain-specific phenomenon governed by motility, *Quorum sensing*, energy metabolism and environmental conditions. The wide variability among isolates emphasises the genetic plasticity of these *Arcobacter* species and its adaptability under diverse conditions, underlining its persistence and relevance as an emerging foodborne pathogen. Nevertheless, gene presence alone did not reliably predict phenotypic capability, stressing the need for functional analyses including gene expression profiling, mutagenesis and transcriptomic approaches.

Limitations of this study include reliance on a single quantitative assay (Christensen method) and the absence of in situ or flow-cell biofilm models, which would better simulate real surfaces and shear forces. Furthermore, although we compared static versus dynamic microtiter plate conditions, in-field conditions in food-processing or aquatic systems may differ markedly. Future work should integrate gene expression data, matrix composition (e.g., EPS, cellulose), surface chemistry and shear dynamics to better define the mechanisms of biofilm resilience in *Arcobacter*.

These data, together with previous findings, indicate that the absence of *flaA*, *flaB*, and *luxS* tends to be associated with reduced biofilm formation, whereas the presence of *spoT* and *fliS* may enhance biofilm formation under defined conditions. The effect of *pta* appears context-dependent, reflecting a balance between metabolic state and biofilm development. Overall, the comparative inclusion of *A. cryaerophilus* confirms that these mechanisms are broadly conserved across the genus *Arcobacter*, though quantitative differences in motility and matrix production highlight subtle but relevant interspecies variability.

## 5. Conclusions

This study provides a comprehensive evaluation of biofilm formation, motility, and the distribution of biofilm-associated genes in 50 *Arcobacter butzleri* and 10 *A*. *cryaerophilus* strains isolated from food and water sources. All strains were found to carry the core biofilm-associated genes *fliS*, *luxS*, *pta*, and *spoT*, whereas *flaA* and *flaB* were present in 70% of the isolates. Similarly, all *A. cryaerophilus* strains possessed the same six biofilm-associated genes, suggesting a highly conserved genetic background related to motility, *Quorum sensing*, and stress response within the genus *Arcobacter*. Notably, even strains lacking these flagellar genes retained motility, demonstrating that alternative mechanisms may support bacterial movement.

Phenotypic analysis using Congo Red Agar identified only a small fraction of cellulose-positive strains, confirming the limitations of this method as a predictor of biofilm formation. In *A. cryaerophilus*, all isolates exhibited the white (CRA-negative) phenotype, despite measurable biofilm formation by quantitative assay, reinforcing that Congo Red Agar is unsuitable for assessing biofilm potential in this species as well. In contrast, quantitative assessment with the Christensen microtiter plate assay revealed that almost all *A*. *butzleri* strains were capable of forming biofilms, with 34% consistently producing strong biofilms under both static and dynamic conditions. All *A. cryaerophilus* isolates also demonstrated biofilm-forming ability, with 70% classified as moderate or strong producers, indicating comparable or slightly lower biofilm intensity compared with *A. butzleri*. Variation in biofilm intensity between cultivation conditions highlighted the influence of environmental factors on biofilm development.

Collectively, these findings demonstrate that biofilm formation is a widespread and strain-dependent trait in *A*. *butzleri* and *A*. *cryaerophilus*. The observed similarities in gene content but subtle interspecific differences in motility and biofilm intensity suggest that while both species share a conserved molecular toolkit for biofilm development, their phenotypic expression may be modulated by species-specific regulation and ecological adaptation. The conserved presence of core biofilm-associated genes, combined with variability in motility and phenotypic biofilm expression, provides a detailed reference framework for understanding the biofilm potential of both *Arcobacter* species. Future investigations should focus on elucidating the regulatory networks controlling biofilm formation, assessing the impact of environmental and stress conditions, and exploring the role of biofilms in the persistence, survival, and potential virulence of *Arcobacter* in food and water environments. These insights will be essential for developing effective strategies to mitigate biofilm-related contamination and enhance food safety.

## Figures and Tables

**Table 1:** Biofilm formation ability of *A. butzleri* strains under static and dynamic conditions, motility, CRA phenotype, and biofilm-associated genes.

Bacterial Strain	Static Cultivation		Dynamic Cultivation		Motility		CRA	Biofilm-Associated Genes
	Absorbance	Category	Absorbance	Category	Mean (cm)	Category		
LMG 10828 *^C^*	0.1261 ± 0.0097	**	0.1372 ± 0.0147	**	1.55 ± 0.05	#	White	*flaA*, *flaB*, *fliS*, *luxS*, *pta*, *spoT*
UPCE 2013/KK *^B^*	0.2134 ± 0.0552	***	0.1683 ± 0.0433	***	0.35 ± 0.05	#	White	*fliS*, *luxS*, *pta*, *spoT*
UPCE 2013/23 *^A^*	0.1297 ± 0.0055	**	0.1281 ± 0.0073	**	0.35 ± 0.05	#	Red	*fliS*, *luxS*, *pta*, *spoT*
UPCE 2013/30 *^B^*	0.3380 ± 0.0931	***	0.2796 ± 0.0736	***	0.65 ± 0.05	#	White	*flaA*, *flaB*, *fliS*, *luxS*, *pta*, *spoT*
UPCE 2015/1 *^B^*	0.1107 ± 0.0018	–	0.1095 ± 0.0030	–	0.40 ± 0.0	#	White	*flaA*, *flaB*, *fliS*, *luxS*, *pta*, *spoT*
UPCE 2015/5 *^A^*	0.1213 ± 0.0041	**	0.1478 ± 0.0052	**	0.30 ± 0.0	#	White	*flaA*, *flaB*, *fliS*, *luxS*, *pta*, *spoT*
UPCE 2015/10 *^B^*	0.3119 ± 0.0219	***	0.2110 ± 0.1163	***	0.50 ± 0.0	#	White	*flaA*, *flaB*, *fliS*, *luxS*, *pta*, *spoT*
UPCE 2015/11 *^B^*	0.1091 ± 0.0020	–	0.1100 ± 0.0052	–	0.45 ± 0.05	#	White	*flaA*, *flaB*, *fliS*, *luxS*, *pta*, *spoT*
UPCE 2015/13 *^B^*	0.2096 ± 0.0050	***	0.1775 ± 0.0091	***	0.30 ± 0.0	#	White	*flaA*, *flaB*, *fliS*, *luxS*, *pta*, *spoT*
UPCE 2015/14 *^B^*	0.1135 ± 0.1086	*	0.1100 ± 0.0345	–	0.45 ± 0.05	#	White	*flaA*, *flaB*, *fliS*, *luxS*, *pta*, *spoT*
UPCE 2015/15 *^B^*	0.2783 ± 0.0200	***	0.3099 ± 0.0165	***	0.50 ± 0.0	#	White	*flaA*, *flaB*, *fliS*, *luxS*, *pta*, *spoT*
UPCE 2015/16 *^B^*	0.2771 ± 0.0422	***	0.2677 ± 0.0306	***	0.50 ± 0.0	#	White	*flaA*, *flaB*, *fliS*, *luxS*, *pta*, *spoT*
UPCE 2015/18 *^B^*	0.1992 ± 0.0652	***	0.1742 ± 0.0072	***	0.30 ± 0.0	#	White	*flaA*, *flaB*, *fliS*, *luxS*, *pta*, *spoT*
UPCE 2015/19 *^B^*	0.1227 ± 0.0022	**	0.1219 ± 0.0022	**	0.55 ± 0.0	#	White	*flaA*, *flaB*, *fliS*, *luxS*, *pta*, *spoT*
UPCE 2019/20 *^B^*	0.1110 ± 0.0029	*	0.1101 ± 0.0284	–	0.50 ± 0.0	#	White	*fliS*, *luxS*, *pta*, *spoT*
UPCE 2021/4 *^B^*	0.1169 ± 0.0037	*	0.1149 ± 0.0024	*	1.60 ± 0.2	#	White	*flaA*, *flaB*, *fliS*, *luxS*, *pta*, *spoT*
UPCE 2021/10 *^B^*	0.1238 ± 0.0078	**	0.1478 ± 0.0148	**	2.15 ± 0.05	##	White	*flaA*, *flaB*, *fliS*, *luxS*, *pta*, *spoT*
UPCE 2021/11 *^B^*	0.1955 ± 0.0233	***	0.2754 ± 0.0116	***	1.90 ± 0.0	#	Red	*flaA*, *flaB*, *fliS*, *luxS*, *pta*, *spoT*
UPCE 2021/12 *^B^*	0.2449 ± 0.0180	***	0.1335 ± 0.0071	**	1.45 ± 0.05	#	White	*fliS*, *luxS*, *pta*, *spoT*
UPCE 2021/13 *^B^*	0.1286 ± 0.0061	**	0.1285 ± 0.0035	**	1.50 ± 0.0	#	White	*flaA*, *flaB*, *fliS*, *luxS*, *pta*, *spoT*
UPCE 2021/15 *^B^*	0.1635 ± 0.0082	***	0.1530 ± 0.0055	***	1.50 ± 0.0	#	White	*flaA*, *flaB*, *fliS*, *luxS*, *pta*, *spoT*
UPCE 2021/32 *^B^*	0.1279 ± 0.0035	**	0.1240 ± 0.0015	**	0.20 ± 0.0	#	White	*flaA*, *flaB*, *fliS*, *luxS*, *pta*, *spoT*
UPCE 2021/33 *^B^*	0.1174 ± 0.0042	*	0.1190 ± 0.0027	*	0.20 ± 0.0	#	White	*flaA*, *flaB*, *fliS*, *luxS*, *pta*, *spoT*
UPCE 2021/41 *^B^*	0.1233 ± 0.0015	**	0.1170 ± 0.0023	*	0.40 ± 0.0	#	White	*fliS*, *luxS*, *pta*, *spoT*
UPCE 2021/42 *^B^*	0.3435 ± 0.1163	***	0.2830 ± 0.0351	***	0.45 ± 0.05	#	White	*flaA*, *flaB*, *fliS*, *luxS*, *pta*, *spoT*
UPCE 2021/43 *^B^*	0.4109 ± 0.1386	***	0.2900 ± 0.0796	***	1.10 ± 0.10	#	White	*flaA*, *flaB*, *fliS*, *luxS*, *pta*, *spoT*
UPCE 2021/44 *^B^*	0.1246 ± 0.0021	**	0.1216 ± 0.0027	**	0.45 ± 0.05	#	White	*fliS*, *luxS*, *pta*, *spoT*
UPCE 2021/45 *^B^*	0.1265 ± 0.0020	**	0.1240 ± 0.0035	**	0.50 ± 0.0	#	White	*flaA*, *flaB*, *fliS*, *luxS*, *pta*, *spoT*
UPCE 2021/46 *^B^*	0.1332 ± 0.0033	**	0.1290 ± 0.0038	**	0.45 ± 0.05	#	White	*fliS*, *luxS*, *pta*, *spoT*
UPCE 2021/47 *^B^*	0.4073 ± 0.0141	***	0.3724 ± 0.2063	***	1.15 ± 0.05	#	White	*flaA*, *flaB*, *fliS*, *luxS*, *pta*, *spoT*
UPCE 2021/48 *^B^*	0.1341 ± 0.0505	**	0.1306 ± 0.0040	**	0.40 ± 0.0	#	White	*fliS*, *luxS*, *pta*, *spoT*
UPCE 2021/49 *^B^*	0.1263 ± 0.0028	**	0.1180 ± 0.0019	*	1.10 ± 0.0	#	White	*flaA*, *flaB*, *fliS*, *luxS*, *pta*, *spoT*
UPCE 24B *^B^*	0.3058 ± 0.0137	***	0.2978 ± 0.0266	***	0.45 ± 0.05	#	White	*flaA*, *flaB*, *fliS*, *luxS*, *pta*, *spoT*
UPCE 26 *^B^*	0.1200 ± 0.0034	**	0.1181 ± 0.0033	*	0.95 ± 0.05	#	White	*flaA*, *flaB*, *fliS*, *luxS*, *pta*, *spoT*
UPCE 30 *^B^*	0.1199 ± 0.0025	*	0.1163 ± 0.0046	*	0.40 ± 0.0	#	White	*flaA*, *flaB*, *fliS*, *luxS*, *pta*, *spoT*
UPCE 43 *^B^*	0.1371 ± 0.0080	**	0.1230 ± 0.0074	**	0.40 ± 0.0	#	Red	*flaA*, *flaB*, *fliS*, *luxS*, *pta*, *spoT*
UPCE 48 *^B^*	0.1141 ± 0.0303	*	0.1174 ± 0.0076	*	0.80 ± 0.1	#	White	*fliS*, *luxS*, *pta*, *spoT*
UPCE 49 *^B^*	0.1183 ± 0.0047	*	0.1180 ± 0.0028	*	0.35 ± 0.05	#	White	*fliS*, *luxS*, *pta*, *spoT*
UPCE 65 *^B^*	0.2509 ± 0.0192	***	0.2243 ± 0.0387	***	0.45 ± 0.05	#	White	*flaA*, *flaB*, *fliS*, *luxS*, *pta*, *spoT*
UPCE 69 *^B^*	0.3351 ± 0.0202	***	0.2846 ± 0.0252	***	2.10 ± 0.0	##	White	*flaA*, *flaB*, *fliS*, *luxS*, *pta*, *spoT*
UPCE 89 *^B^*	0.1237 ± 0.0013	**	0.1176 ± 0.0033	*	0.50 ± 0.0	#	White	*fliS*, *luxS*, *pta*, *spoT*
UPCE 93 *^B^*	0.1169 ± 0.0031	*	0.1118 ± 0.0021	*	1.10 ± 0.0	#	Red	*flaA*, *flaB*, *fliS*, *luxS*, *pta*, *spoT*
UPCE 94 *^B^*	0.1208 ± 0.0030	**	0.1172 ± 0.0029	*	1.30 ± 0.0	#	White	*fliS*, *luxS*, *pta*, *spoT*
UPCE 107 *^B^*	0.1240 ± 0.0035	**	0.1132 ± 0.0030	*	0.40 ± 0.0	#	White	*fliS*, *luxS*, *pta*, *spoT*
UPCE 132 *^B^*	0.1184 ± 0.0034	*	0.1129 ± 0.0036	*	0.35 ± 0.05	#	White	*flaA*, *flaB*, *fliS*, *luxS*, *pta*, *spoT*
UPCE 134 *^B^*	0.3333 ± 0.0261	***	0.2767 ± 0.0449	***	1.0 ± 0.0	#	White	*fliS*, *luxS*, *pta*, *spoT*
UPCE 138 *^B^*	0.1606 ± 0.0105	***	0.1569 ± 0.0129	***	1.70 ± 0.0	#	White	*fliS*, *luxS*, *pta*, *spoT*
UPCE 141 *^B^*	0.1192 ± 0.0053	*	0.1211 ± 0.0052	**	0.85 ± 0.05	#	Red	*flaA*, *flaB*, *fliS*, *luxS*, *pta*, *spoT*
UPCE 160 *^B^*	0.1178 ± 0.0039	*	0.1209 ± 0.0030	**	0.30 ± 0.0	#	White	*flaA*, *flaB*, *fliS*, *luxS*, *pta*, *spoT*
UPCE 161 *^B^*	0.1707 ± 0.0119	***	0.1279 ± 0.0057	**	0.50 ± 0.0	#	White	*flaA*, *flaB*, *fliS*, *luxS*, *pta*, *spoT*

*^A^* Strain isolated from water; *^B^* Strain isolated from food, *^C^* Type strain from the Belgian Coordinated Collections of Microorganisms (BCCM/LMG); UPCE—Strains isolated at University of Pardubice; CRA—phenotype on Congo Red Agar. – non-adherent, * weakly adherent, ** moderately adherent, *** strongly adherent, # low motility, ## moderate motility.

**Table 2:** Biofilm formation ability of *A. cryaerophilus* strains under static and dynamic conditions, motility, CRA phenotype, and biofilm-associated genes.

Bacterial Strain	Static Cultivation		Dynamic Cultivation		Motility		CRA	Biofilm-Associated Genes
	Absorbance	Category	Absorbance	Category	Mean (cm)	Category		
CCM 3933 *^C^*	0.1280 ± 0.0088	**	0.1198 ± 0.0058	*	0.45 ± 0.08	#	White	*flaA*, *flaB*, *fliS*, *luxS*, *pta*, *spoT*
UPCE 2013/13 *^A^*	0.1412 ± 0.0109	**	0.1614 ± 0.0325	***	1.05 ± 0.05	#	White	*flaA*, *flaB*, *fliS*, *luxS*, *pta*, *spoT*
UPCE 2015/58 *^A^*	0.1262 ± 0.0099	**	0.1285 ± 0.0085	**	0.40 ± 0.07	#	White	*flaA*, *flaB*, *fliS*, *luxS*, *pta*, *spoT*
UPCE 2015/59 *^B^*	0.1812 ± 0.0242	***	0.2015 ± 0.0514	***	0.50 ± 0.04	#	White	*flaA*, *flaB*, *fliS*, *luxS*, *pta*, *spoT*
UPCE 2015/51 *^B^*	0.1638 ± 0.0143	***	0.1252 ± 0.0041	**	0.85 ± 0.10	#	White	*flaA*, *flaB*, *fliS*, *luxS*, *pta*, *spoT*
UPCE 2015/52 *^A^*	0.1586 ± 0.0229	***	0.1410 ± 0.0075	**	0.40 ± 0.10	#	White	*flaA*, *flaB*, *fliS*, *luxS*, *pta*, *spoT*
UPCE 2015/54 *^B^*	0.1460 ± 0.0101	**	0.1614 ± 0.0785	***	0.85 ± 0.05	#	White	*flaA*, *flaB*, *fliS*, *luxS*, *pta*, *spoT*
UPCE 2015/55 *^B^*	0.1144 ± 0.0049	*	0.1100 ± 0.0085	–	0.75 ± 0.08	#	White	*flaA*, *flaB*, *fliS*, *luxS*, *pta*, *spoT*
UPCE 2015/56 *^B^*	0.1117 ± 0.0034	*	0.1215 ± 0.0043	**	0.60 ± 0.07	#	White	*flaA*, *flaB*, *fliS*, *luxS*, *pta*, *spoT*
UPCE 2015/57 *^B^*	0.1251 ± 0.0047	**	0.1158 ± 0.0074	*	1.50 ± 0.07	#	White	*flaA*, *flaB*, *fliS*, *luxS*, *pta*, *spoT*

*^A^* Strain isolated from water; *^B^* Strain isolated from food, *^C^* Type strain from the Czech Collections of Microorganisms (strain identical to the reference strain ATCC 43158^T^); UPCE—Strains isolated at University of Pardubice; CRA—phenotype on Congo Red Agar. – non-adherent, * weakly adherent, ** moderately adherent, *** strongly adherent, # low motility.

## Data Availability

The original contributions presented in this study are included in the article. Further inquiries can be directed to the corresponding author.
